# New nematogenic conical-shaped supramolecular H-bonded complexes for solar energy investigations

**DOI:** 10.1038/s41598-021-97126-5

**Published:** 2021-09-02

**Authors:** Sobhi M. Gomha, Hoda A. Ahmed, Mohamed Shaban, Tariq Z. Abolibda, Khalid Abdulaziz Alharbi, Hafsa H. Alalawy

**Affiliations:** 1 Chemistry Department, Faculty of Science, Islamic University of Madinah, Al-Madinah Al-Munawwarah, 42351 Saudi Arabia; 2 Department of Chemistry, Faculty of Science, Cairo University, Giza, 12613 Egypt; 3 Chemistry Department, College of Sciences, Yanbu, Taibah University, Yanbu, 30799 Saudi Arabia; 4 Department of Physics, Faculty of Science, Islamic University of Madinah, Al-Madinah Al-Munawwarah, 42351 Saudi Arabia

**Keywords:** Chemistry, Energy science and technology, Materials science, Optics and photonics, Physics

## Abstract

New conical-shaped geometrical supramolecular H-bonded liquid crystal complexes were formed through 1:2 intermolecular interactions of H-bonding between flexible core (adipic acid, **A**) and lateral chloro-substituted azopyridines (**Bn**). The chains of the terminally alkoxy substituted base (**n**) were changed between 8 and 16 carbons. Mesomorphic and optical examinations of the prepared complexes were measured via differential scanning calorimetry (DSC) and polarizing optical microscopy (POM). Fourier-transform infrared spectroscopy (FT-IR) was used to confirm the Fermi bands of the H- bonding interactions. Induced nematogenic mesophases that cover the whole lengths of alkoxy-chains were detected. The non-linear geometries of the designed supramolecular complexes were also confirmed via Density functional theory (DFT) calculations. It was found that the length of terminal alkoxy chain of the base moiety highly affects the geometrical structure of the investigated complexes. Moreover, it increases the thermodynamic energy and influences the geometrical parameters. The electrical properties of each of the acid component (**A**), the base (**B16**) and their 1:2 complex (**A/2B16**) were evaluated using the Keithley measurement-source unit. The optical properties studies showed that the influences in the optical absorption and the reduction of the energy gap of the complex compared to its individual components made the resulted supramolecular H-bonded complex soft material suitable for solar energy investigations.

## Introduction

Liquid crystalline (LC) materials are considered as a type of functional compounds with great potentials having the molecular order and mobility to be used in display applications^1^. In recent years, molecular interactions resulting from hydrogen bonding (H-bonding) LC mixtures^2–6^ have received additional interest. Early examples of the supramolecular hydrogen bonded liquid crystal (SMHBLC) systems were reported by Gray et.al.^7^, who studied the thermal characterizations of 4-n-alkoxybenzoic acids. Mesomorphic properties of the alkoxy-substituted benzoic acids were attributed to the formation of calamitic symmetric mixtures of interacted benzoic acid molecules forming a supramolecular core via hydrogen bonding interactions^7,8^. Recently, this approach was extended to the formation of new geometrical architectures of supramolecular complexes as well as new twist-bended nematogens (bent SMHBLCs)^9^. The supramolecular mesogens formation via hydrogen bonding interactions is generally more efficient than covalent bonding, and a new approach of introducing its functionality within the molecular skeleton, in an effective and controllable manner, has been established^10^.

LC-based based azobenzene derivatives are being studied due to their cooperative interactions of mesogenic moieties which lead to induced photoanisotropy property. Introduction of the azobenzene moiety into the molecular structure of LC materials resulted in photo-switchable LCs^11,12^. Also, azo compounds have extended a variety of biological activities^13–15^, optical-switches, and non-linear optics (NLO)^1,16–21^. In addition, they have been used as absorbing dyes molecules^22^ due to their ability for molecular trans/cis photo-isomerization when irradiated with UV light^23^. Moreover, pyridines are a group of important chemical compounds due to their biological activities^24–26^. It has been recently reported that pyridine containing compounds have many applications in perovskite solar cells^27–30^.

On the other hand, terminal groups have essential roles in the stability and mesophase temperature range of the prepared LC compounds. As the length of the terminal alkyl or alkoxy chains increases, the molecules tend to orient in parallel arrangements^31^, which enhances the observation of the mesophase. Likewise, lateral substituents have essential impact in the reduction of melting transition temperatures, changing the mesomorphic ranges and their thermal stability, as well as the predominance of the nematic phase^32,33^.

The introduction of lateral-substituents in the mesogenic portion of rod-like LC molecules has been widely documented, and all of its results have accumulated in the change of thermal and optical behaviors of the LC compounds, depending on the type, orientation, and position of the substituent^33–35^. Furthermore, the theoretical calculations have been used to predict the geometrical and thermal parameters of liquid crystalline molecules and their complexes^36,37^.

Band-gap engineering and optical properties control are very important parameters for solar energy investigations^38–42^. Organic materials based azo derivatives are used in many applications such as displays, solar cells, sensors, modulators, etc.^43^. In general, crystalline organic compounds display better transportation properties than their polymeric equivalents. Many documented researches investigated the photovoltaic impacts in symmetrical cells filled with optical organic materials^44–46^. Photovoltaic impact analogous to that of some of the better organic solar cells was reported^46–48^.

**Scheme 1.**
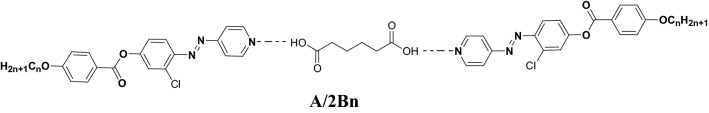
1:2 SMHBCs, **A/ 2Bn**.

In order to study further the factors governing the mesomorphic and thermal behaviors of supramolecular liquid crystal complexes and the relationship with their molecular structures, symmetrical 1:2 supramolecular H-bonded complexes based on adipic acid core were considered^26,49^. Herein, a new homologues of the lateral chloro azo/ester derivatives **A/2Bn** are prepared through H-bonding interactions between the flexible adipic acid (**A**) and the laterally Cl substituted azopyridine base derivatives (**Bn**), aiming to study the effect of the lateral electron withdrawing (Cl) group on the formation, type and stability of the mesophases, as well as on the predicted optimized geometrical calculations and to investigate its experimental and theoretical relationship. The impact of terminal alkoxy length of the base fragments on the thermal, electrical, and optical properties of the prepared complexes (**A/2Bn**) is also investigated. The optical band-gap energy and band-tail are measured as a function of the terminal lengths. Furthermore, comparisons are to be conducted between the present lateral Cl complexes and previously studied laterally CH_3_ substituted analogues and their laterally neat SMHB complexes to study the impact of the kind of lateral substituent on the mesophase behaviour (Scheme 1).

## Experimental

4-(2-(Pyridin-4-yl)diazenyl-(3-chlorophenyl) 4-alkoxybenzoate (**Bn**) were synnthesized according to the reported method^50^, attached in supplementary materials.

### Preparation of H-bonded complexes (1:2)

**Scheme 2.**
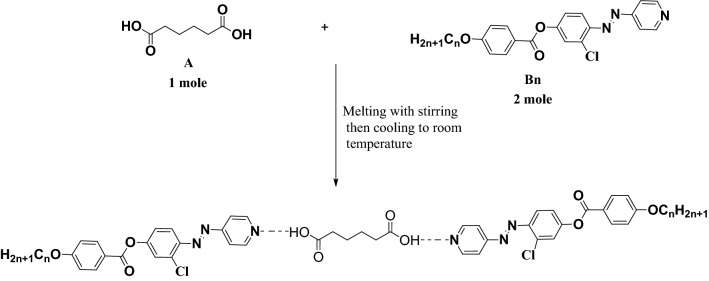
Formation of present complexes (**A/2Bn**).

The SMHBCs (**A/2Bn**) were prepared from adipic acid (**A** , one mole) and 4-(2-(pyridin-4-yl)diazenyl-(3-chlorophenyl) 4-alkoxybenzoate (**Bn**, two moles), with n varying between n = 8 to n = 16 carbons. The solid mixture was melted with stirring to prepare an intimate blend and then allowed to cool to room-temperature ( see Scheme 2).

## Results and discussion

### FT-IR confirmation of prepared complexes

**Figure 1:**
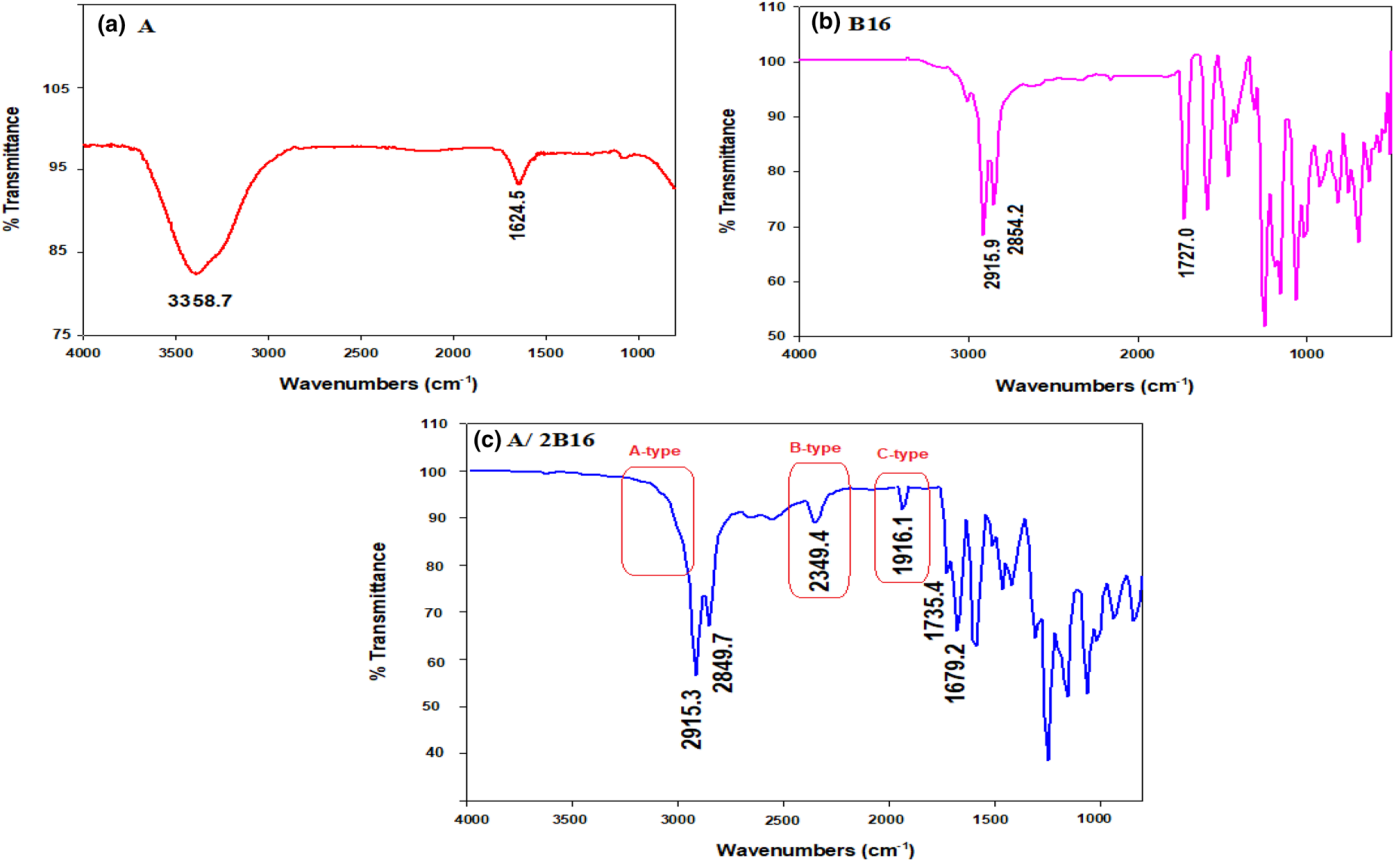
FT-IR spectra of (**a**) Adipic acid, **A**; (**b**) Azopyridine, **B16** and (**c**) their supramolecular H-bonded complex **A/2B16**.

FT-IR spectroscopy was applied to confirm the formation of H-bonded interactions in the prepared complexes (**A/2Bn**) between the adipic acid (**A**) and the lateral-chloro azopyridines (**Bn**). FT-IR investigations were performed for the individual components and their 1:2 Supramolecular H-bonded complexes. Figure 1 displays the FT-IR spectrum of adipic acid (**A**), lateral chloro azopyridine homologue (**B16**), and their H-bonded complex **A/2B16** as representative examples. FT-IR spectrum of **B16** shows an intense band at 1727.0 cm^−1^ related to the presence of an ester carbonyl group (COOAr) which increases to the less intense band at 1735.4 cm^−1^ upon complex formation. Also, complexation leads to shifting of the carboxylic carbonyl (COOH) peak of adipic acid from 1624.5 to 1679.2 cm^−1^. These shifts in measurments confirm the intermolecular H-bond formation. Furthermore, three fermi-resonant vibration band observations of the H-bonded A, B, and C types also support H-bond formation^51–55^. The **A-type** Fermi-band of the complex **A/2B16** overlapps at 2915.3 and 2849.7 cm^−1^ with that of the C-H vibrational peaks. The peak observed for the complex at 2349.4 cm^−1^ could be assigned to the B-type of the O–H group in-plane bending vibration. Further, the interaction between the fundamental stretching vibration of the OH group and the overtone of the torsional effect produces a band at 1916.1 cm^−1^ correspondent to the **C-type** Fermi-band.

### Mesomorphic and optical studies

**Figure 2:**
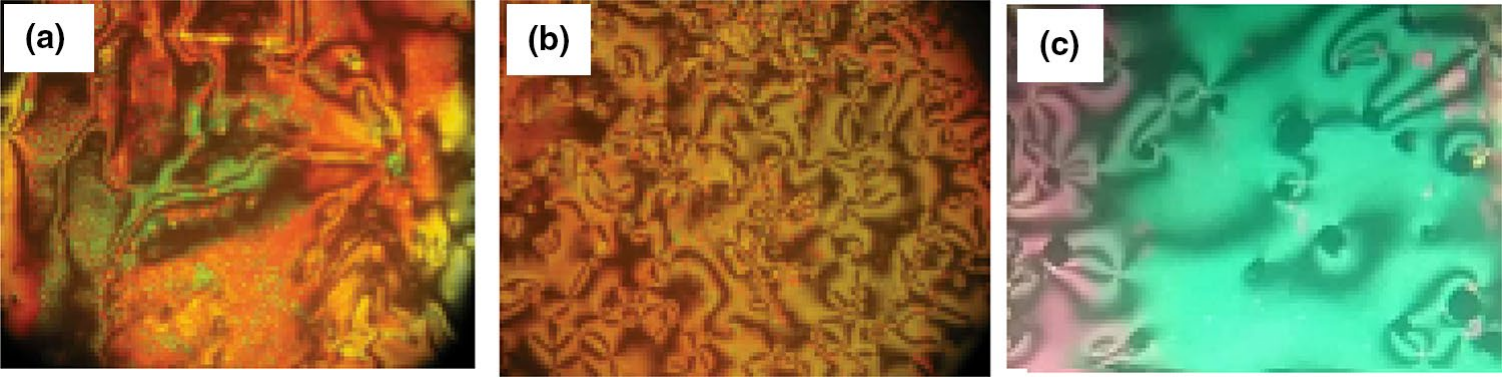
Schlieren nematic mesophases via heating under POM for (**a**) **A/2B8** at 111.0 °C (**b**) **A/2B12** at 104.0 °C and (**c**) **A/2B10** at 108.0 °C.

**Figure 3:**
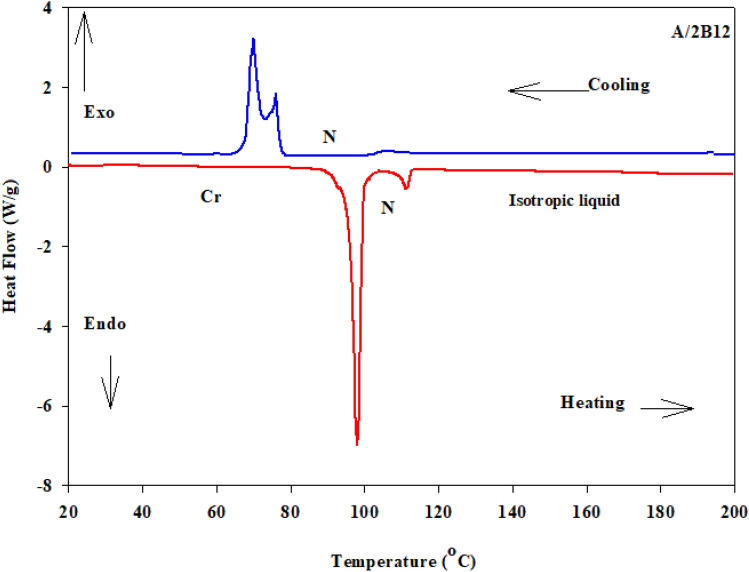
DSC thermograms on heating/cooling rounds with heating rate 10 °C/min of present complex **A/2B12**, as representative example.

**Figure 4:**
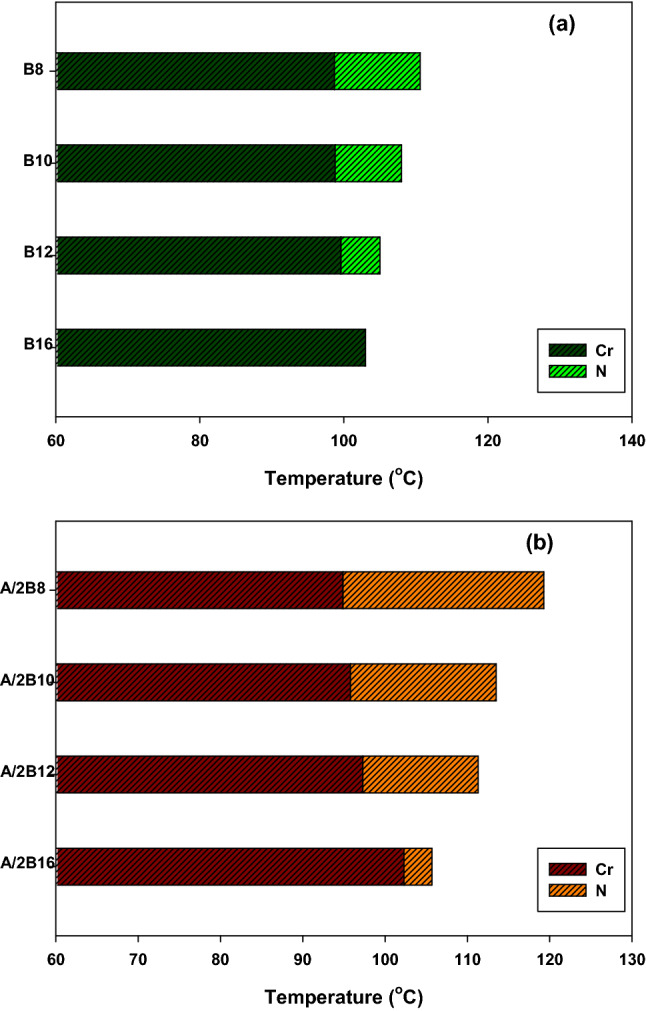
Graphical DSC transitions of (**a**) azopyridine homologues series (**Bn**) and (**b**) their supramolecular H-bonded complexes **A/2Bn** from second heating cycle.

**Figure 5:**
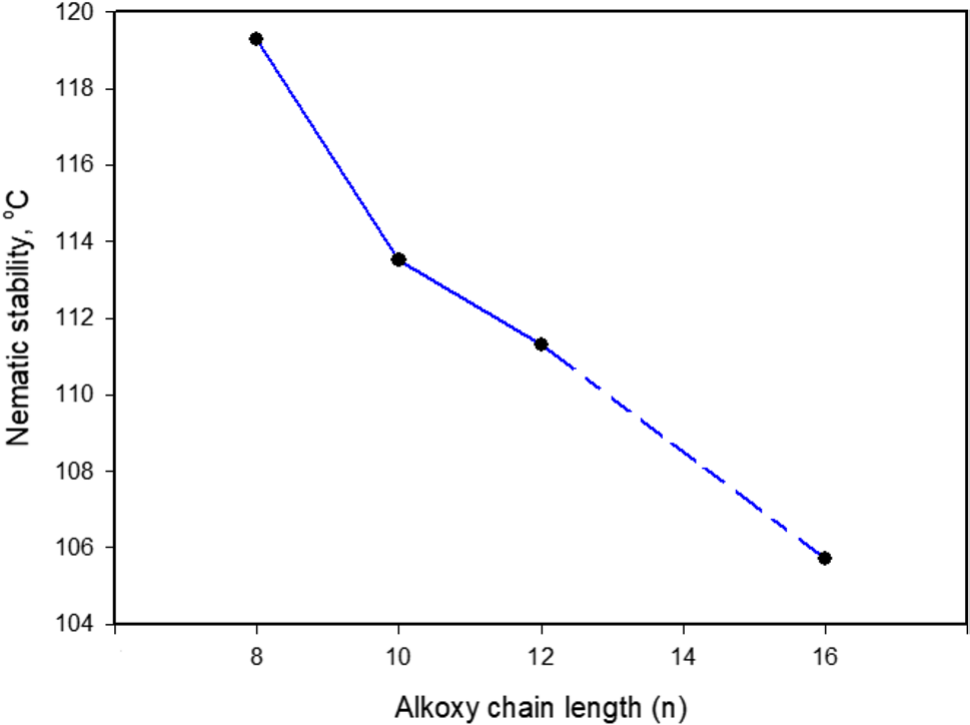
Dependence of nematic stability (***T***_N−I_) on the terminal alkoxy chain-length (n).

Mesogenic transitions and optical properties of the formed supramolecular H- bonded complexes (A/2Bn) were investigated by DSC and POM instrumentations. Mesophase transitions, as derived from DSC analysis, for all designed complexes are summarized in Table 1. Images of the observed phases were verified by POM and they confirmed the nematogenic schlieren textures as given in Fig. 2. Nematic mesophase was only observed upon both rounds of heating and cooling for all complexes. DSC thermograms upon heating and cooling cycles of the studied **A/2B12** complex, as representative example, are displayed in Fig. 3 upon the second heating/cooling rounds. The thermogram, in Fig. 3, shows in the heating cycle two endothermic peaks and in the cooling cycle also two exothermic peaks. These two transitions observed in the heating cycle correspond to the melting and nematic to isotropic transition upon heating, and the reversed isotropic refer to nematic mesophase and nematic to crystalline upon cooling. All formed supramolecular complexes (**A/2Bn**) showed good thermal stabilities of enantiotropic nematic phase (N). In order to investigate the effect of the terminal alkoxy chain length on the mesogenic behavior of azopyridines (**Bn**)^23^ and SMHB complexes a graphical representation of the transition temperature is illustrated in Fig. 4. The most stable geometric shapes of the prepared complexes will be investigated later in the computational part. The mesomorphic and optical behaviors are mainly dependent on the kind and length of terminal flexible groups. Resulted values in Table 1 and Fig. 4b reveal that only the enantiotropic N phase is formed in all of the investigated 1:2 molar SMHBCs (**A/2Bn**) and their thermal stabilities decrement with increasing the length of terminal alkoxy base chain (n) is in agreement with previous documents^56,57^ (See Fig. 5). These results may be attributed to the stacking of the aromatic rings strength together with the aggregation of the terminal alkoxy chains. The stacking of the aromatic rings is more pronounced than the aggregation of the chains. By comparing the transition behavior of azopyridens (Fig. 4a) and the newly formed complexes (Fig. 4b), it was found that an induced range of nematogenic phase was observed due to complexation and it covered all mixtures. It has been found that the polarity difference between H-donors and H-acceptor components of the complexes affects the H-bonding strength and consequently influences the molecular anisotropy thus promoting broadening of the mesophase range^58^. However, the polarity of both complementary components of the mixture is not impacted by the length of the terminal alkoxy chain. The study revealed that the melting transitions (T_Cr–N_) of present complexes exhibit irregular trends. The data also revealed that the complex **A/2B16** possesses the highest melting temperature (102.3 °C) while the homologue **A/2B8** exhibits the lowest meting point (94.9 °C). The investigation also shows that the nematic range (∆T_C_) linearly decreases with terminal length (*n*) in the order: **A/2B8** > **A/2B10** > **A/2B12** > **A/2B16**. The complex **A/2B8** exhibits an enhanced nematic mesophase with nematic temperature range, ∆T_C_ = 24.4 °C. Whereas, the remaining homologues **A/2B10**, **A/2B12**, and **A/2B16** exhibit N mesophases with nematic temperature ranges 17.7, 14.0 and 3.4 °C, respectively.

**Table 1:** Temperature of phase transition (°C), enthalpy of transitions (kJ/mol), normalized entropy and the nematic temperature range (***∆T***_C_) for the present complexes **A/2Bn**.

Complex	*T*_Cr-N_	*∆H*_Cr-N_	*T*_N-I_	*∆H*_N-I_	*∆S/R*_N-I_	*∆T*_C_
A/2B8	94.9	87.63	119.3	4.17	1.28	24.4
A/2B10	95.8	92.95	113.5	3.51	1.09	17.7
A/2B12	97.3	94.85	111.3	3.46	1.08	14.0
A/2B16	102.3	82.56	105.7	1.84	0.58	3.4

*Cr–N* crystal-nematic phase; *N-I* nematic-isotropic liquid. ***∆T***_C =_ Nematic range.

**Figure 6:**
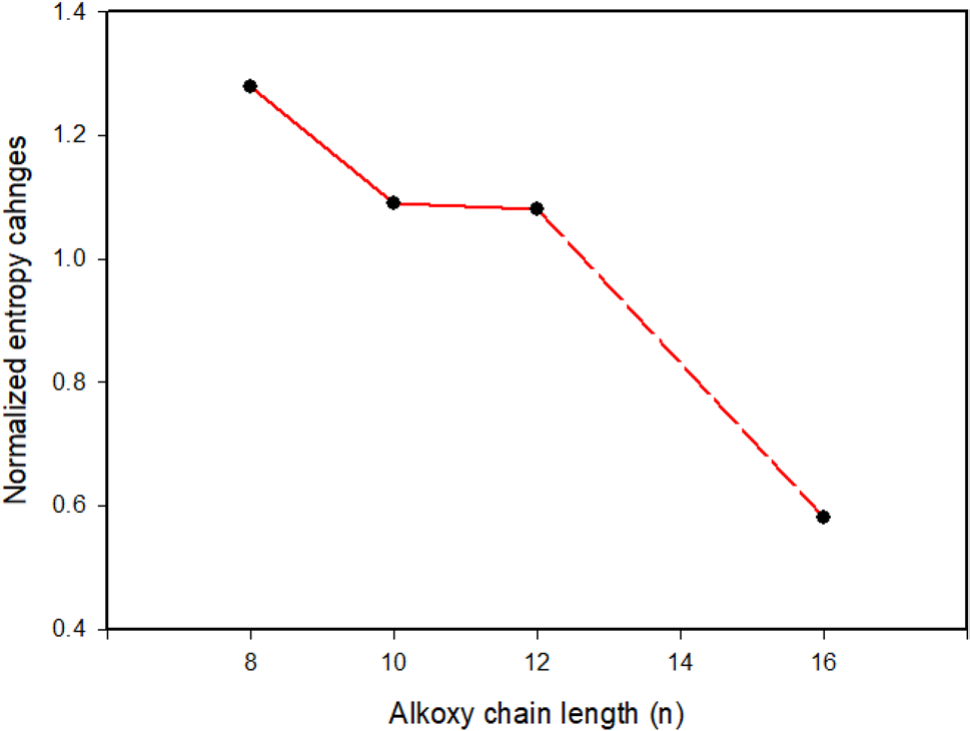
Dependence of entropy changes on the terminal alkoxy chain length (n).

Table 1 tabulates the crystal-to-nematic transition temperatures (***T***_Cr-N_) and their enthalpies (***∆H***_Cr-N_), entropies, and their respective nematic-to-isotropic transitions (***T***_N-I_, ***∆H***_N-I_) as well as the entropy of nematic transition (***∆S/R***_N-I_) of the present complexes **A**/2**B***n*, derived from their DSC measurements. The resulted entropy change (***∆S/R***_N-I_) decreases linearly with the increments of the alkoxy chain *n* (Fig. 6). The formation of the less ordered N phase is attributed to the increment of the molecular end-end aggregations with lengthening the base terminal alkoxy chains.

### Molecular geometries and theoretical studies

**Figure 7:**
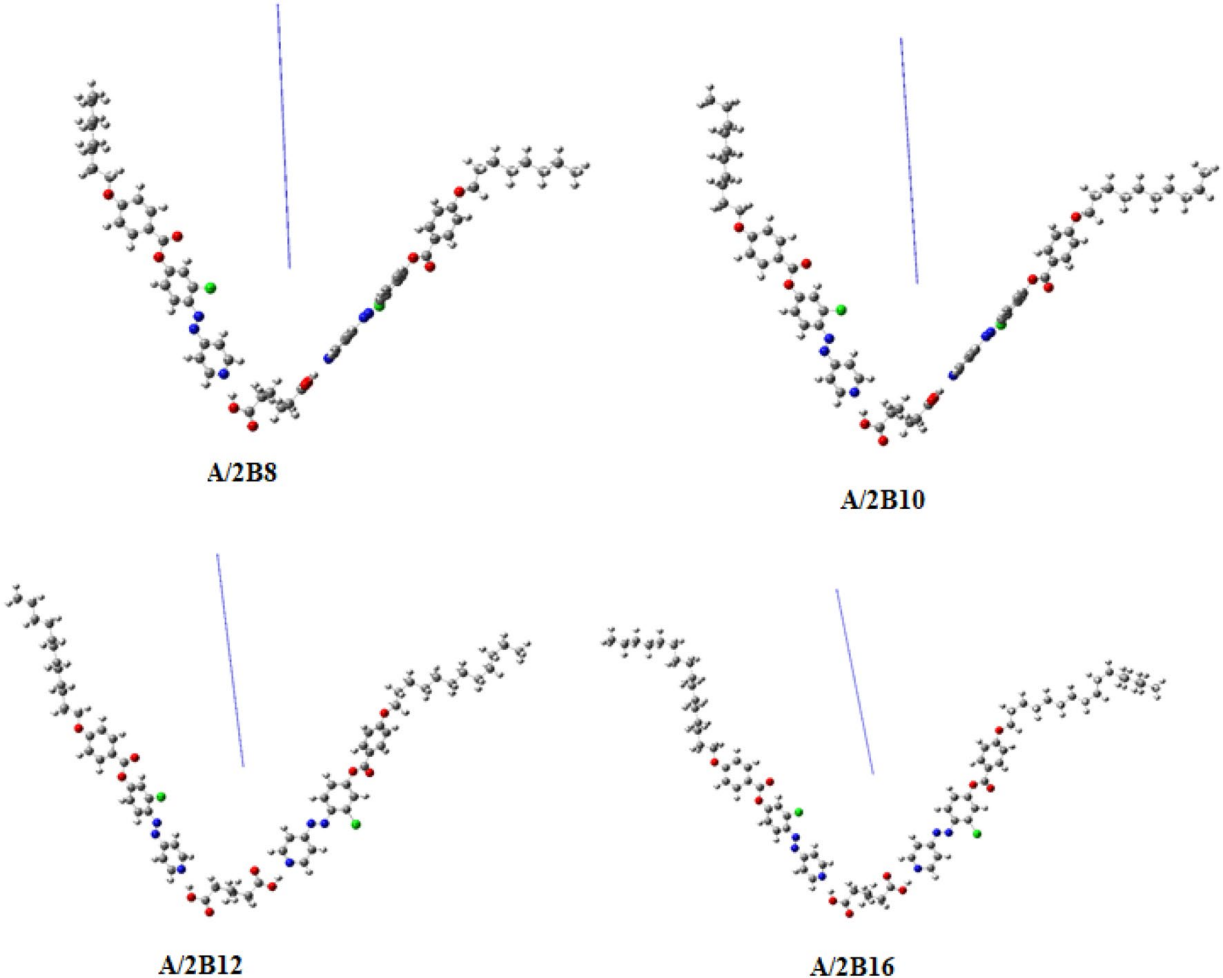
Atomic charges and dipole moment vector for the optimized structures of prepared complexes A/2Bn, calculated at B3LYP/6-31 g(d,p) level.

Our studies were constructed between the theoretical quantum chemical parameters, measured by DFT method, and the experimental findings for the present supramolecular complexes **A/2Bn**. The computational calculations were carried out in the gas phase at B3LYP level using 6-31G (d,p) as the basis set of predicted geometrical shapes. All calculations were performed by the Gaussian 09 W package^59^. As can be seen from Fig. 7, all investigated H-bonded complexes are non- linear having conical-shaped geometries despite the fact that both components of the mixture are linear with planar geometry. As shown from Fig. 7, the right side of the H-bond is shorter than the left side H-bond with approximately close values for the four complexes. The conical shape is manifested by the dihedral angle between the central 4 carbon atoms (see Table S1 and Figure S1 in supplementary data) with an absolute value around 68° and 70°. As shown from Fig. 7, the length of terminal alkoxy chain of the base moiety highly impacts the geometrical structure of the complex.

The estimated thermal parameter values of the theoretical predictions are collected in Table 2. These data were correlated with the experimental results of the mesomorphic transitions as well as the length of azopyridine terminal alkoxy chains (*n*). It was concluded from the results that the polarity, polarizability, aspect ratio, rigidity and geometry of the attached substituents on the mesomorphic molecules are essential parameters influencing the type and thermal stability of the formed mesophases^56^. In addition, the mesomorphic properties were found to be intensely dependent on the length of the terminal chains which is the most often assigned term of molecular shape^6^. The competition between intermolecular lateral and terminal molecular interactions affects the mesophase behaviour. This competition of the two types of interaction leads to the predomination of one of them due to the structural optimization. The conical-shape of the present H-bonded complexes enhances the terminal interactions over the end-to-end interactions. This consequently resulted in the formation of the less ordered phase (nematic mesophase) in all mixtures and being predominant. As can be seen from Table 2, a slight decrease of dipole moment is noticed as the length of alkoxy base chain (*n*) increases from *n* = 8 to *n* = 16 carbons. Ionization energy and electron affinity can be calculated as I.E = -E_HOMO_ and E.A = -E_LUMO_, respectively^60^. The observed decrease of the ionization energy, I.E, is related to the increased ability to undergo oxidation, i.e. loss of electrons and forming a cation, while the electron affinity, E.A, is related to stability and chemical reactivity^61^.

**Table 2:** Total Energy**,** E_HOMO_, E_LUMO_, ∆E, dipole moment, ionization energy, electron affinity and softness parameter calculated at B3LYP/6-31 g(d,p) level for the H-bonded complexes, **A/2Bn.**

Complex	Total ENERGY, Hartree	E_HOMO_, eV	E_LUMO_, eV	∆E, eV	Dipole moment, Debye	I.E, eV	E.A, eV	Global softness S = 1/∆E
A/2B8	− 4251.182	− 6.145	− 2.991	3.155	20.80	6.145	2.991	0.317
A/2B10	− 4408.445	− 6.149	− 2.988	3.161	20.53	6.149	2.988	0.316
A/2B12	− 4565.712	− 6.323	− 3.004	3.318	20.07	6.323	3.004	0.301
A/2B16	− 4880.242	− 6.333	− 3.002	3.331	19.64	6.333	3.002	0.300

*E*_*HOMO*_ energy of the highest occupied molecular orbital, *E*_*LUMO*_ energy of the lowest unoccupied molecular orbital and ∆E = E_LUMO_ − E_HOMO_; orbital energy gap.

**Figure 8:**
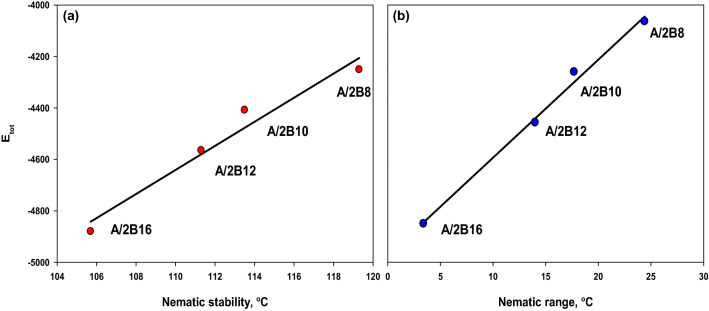
Correlation between the sum of the electronic & themal energies of present complexes **A/2Bn** with their (**a**) nematic mesophase stability and (**b**) nematogenic range in the conical-shaped geometry.

Figure 8 shows the correlation between the sum of the electronic & themal energies of investigated complexes (**A/2Bn**) and their nematic temperature range and stability. As can be seen from Table 2 and Fig. 6, there is a pronounced decrease in the thermal energies with the increment of nematic temperature range and stability as the length of alkoxy terminal chain (**n**) increases (Fig. 8a,b). Thus, lengthening of the terminal chain of the azopyridine component of the mixture highly enhances the estimated thermal stability of the resulting complexes. The longer the chain length (**n** = 16) the more the Van der Waal aggregation of the alkoxy chains, hence it becomes lower in the predicted total energy (-4880.242 Hartree). The results showed that the strength of the terminal aggregation increases as the chain length of alkoxy base increases, with a decrease in the total thermodynamic energy and in the N mesophase stability.

#### Frontier molecular orbitals

**Figure 9:**
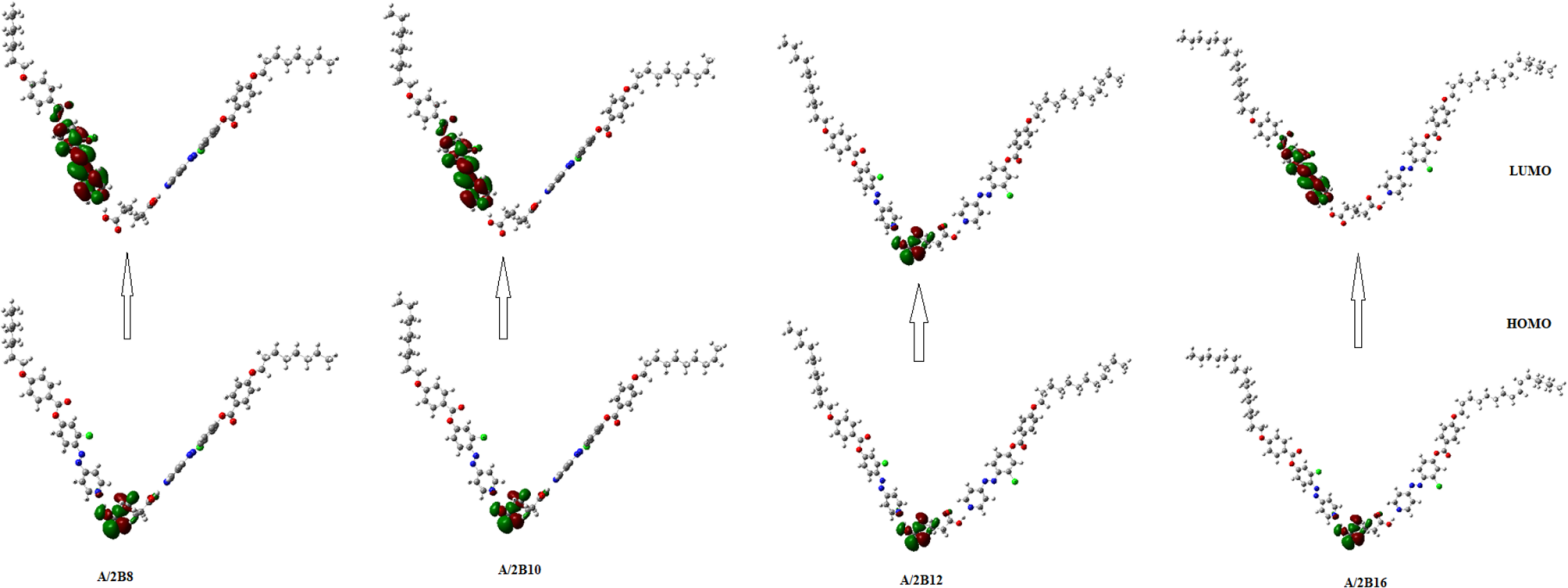
Predicted geometry for FMOs of conical-shaped series, **A/2Bn**.

For the estimated conical-shaped geometrical structures of prepared complexes (**A/2Bn**)**,** the frontier molecular orbitals HOMO (highest occupied) and LUMO (lowest unoccupied) diagrams are displayed in Fig. 9**.** Their resulting energies as well as energy gap (∆E) are collected in Table 2. The prediction of the tendency of electron transfer from HOMO to LUMO during electronic excitation mechanism is assigned by the energy gap and it is inversely related to reactivity^55,62^. The estimated data revealed that, the electron densities of the sites that contributed in the formation of the HOMOs and the LUMOs are localized upon the azo-linkage. Additionally, there is a slight effect of the short terminals of the azopyridine moiety (n = 8 and 10) on the location of the electron densities of the FMOs. Whereas, the longer terminal chain (*n* = 16) exhibits the lowest values of electron density. On the other hand, the energy gap of FMOs is linearly dependent on the length of the alkoxy chain (**n**). The calculated global softness (**S**) is also included in Table 2. This parameter is used to predict the polarizability and sensitivity of the materials for the photoelectric effects. The high values of **S** observed for the compounds indicate their photoelectric sensitivity as well as their polarizability. As shown from Table 2, the lower homologues, **A/2B8** and **A/2B10** have higher global softness than either of their higher homologues **A/2B12** and **A/2B16**.

On the other hand, their lower energy gaps have led to the increment in their polarizability. Moreover, the dipole moment is an important parameter that impacts the mesomorphic behavior of prepared materials. From Table 2**,** the dipole moment of the conical-shaped complexes linearly decreases with the terminal alkoxy chain length of the base component. The higher dipole moment of **A/2B8** complex increases their nematic stability and temperature range.

#### *Molecular electrostatic potential* (*MEP*)

**Figure 10:**
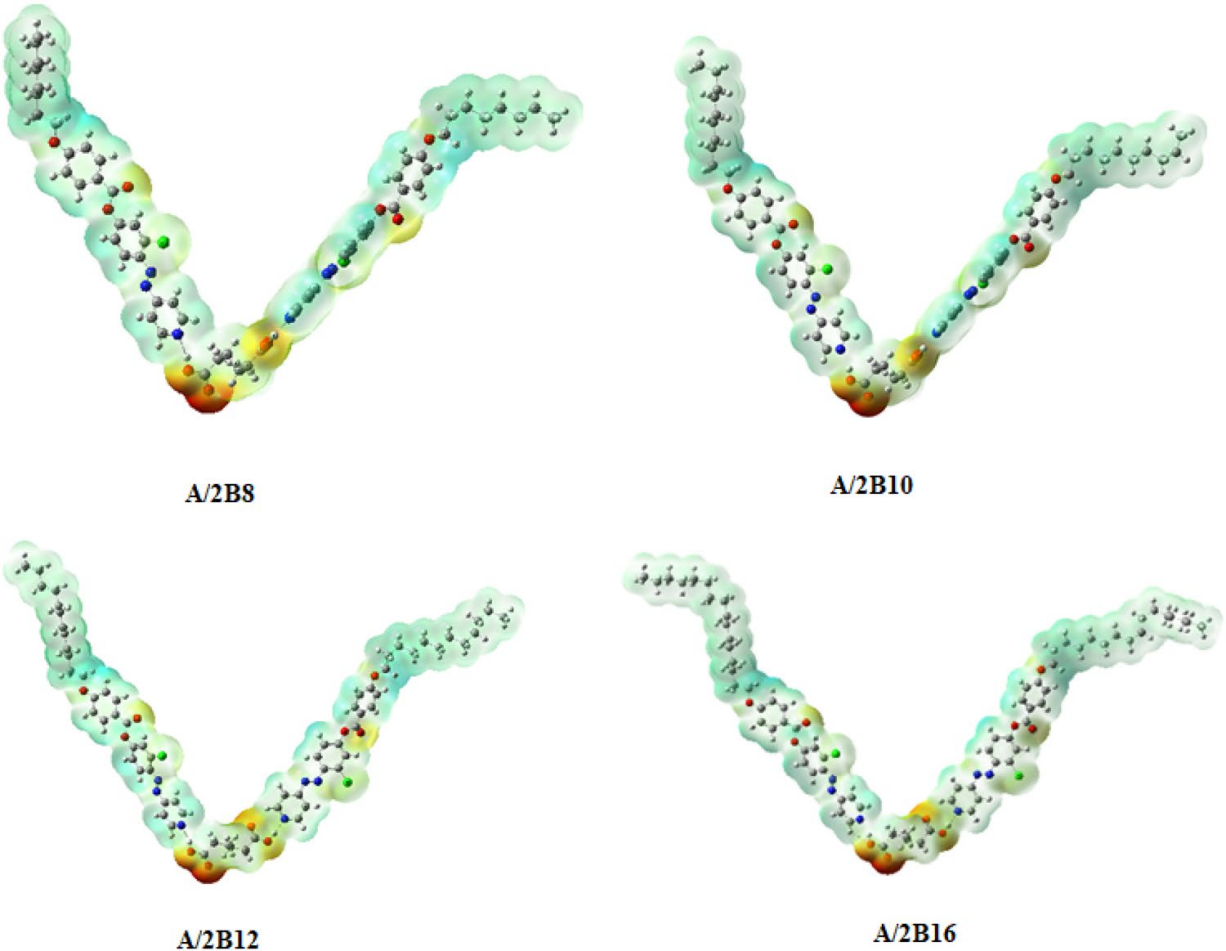
MEP for present H-bonded complexes, **A/2Bn**.

The charge distribution map for the prepared complexes (**A/2Bn**) was estimated at the same level of theory by the same basis sets according to MEP (Fig. 10). Red regions, assigned to the negatively charged atomic sites, were estimated to be localized upon the H-bonded carboxylate moiety of the adipic acid component. The alkoxy chains of azopyridens were predicted to show blue regions of the least negatively charged atomic sites. Figure 10 also shows that the position of lateral chloro substituent in the base component influences the amount of charges. This may be attributed to the conical-shaped architecture of the complex which facilitates the terminal aggregations to influence the nematic phase formation for all lengths of the terminal chains. This molecular geometry permits the maximum terminal alkoxy chain interactions to observe the N phase in all of the prepared complexes.

### Electrical properties

**Figure 11:**
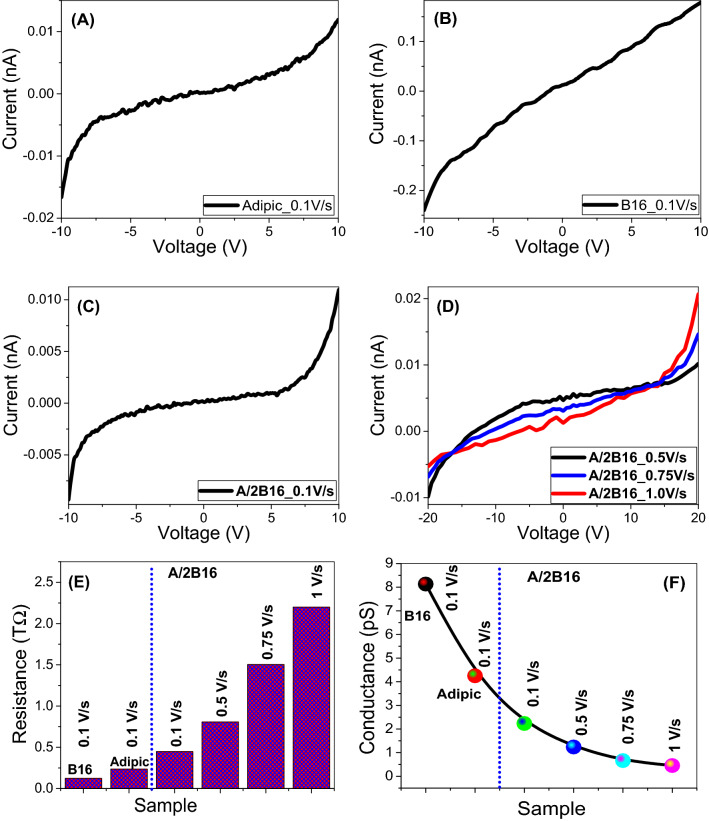
Current–voltage characteristics of (**A**) Adipic acid, **A**; (**B**) Azopyridine, **B16** and (**C**) their supramolecular H-bonded complex **A/2B16**; (**D**) current–voltage characteristics of **A/2B16** at different scan rate; the obtained values of (**E**) resistance and (**F**) conductance of **A, B16**, and **A/2B16** at different scan rates.

The electrical properties of the investigated samples are analyzed using the Keithley measurement source unit ( Model 2400 SMU). The current–voltage (*I–V*) response of the adipic acid (**A**), azopyridine base (**B16**)**,** and their supramolecular complex **A/2B16** films are measured via changing the applied voltage (V) from − 10 V to 10 V with a step of 0.1 V, as shown in Fig. 11A–C. It is evident that the behaviors are non-Ohmic (non-linear), which means that the resistance of the materials changes based on the current moving through it. Recent works showed that the polymeric and organic systems are of Schottky diode behavior at low voltage. But in the present evaluation, the relation between log (I) and V^1/2^ is non-linear, as illustrated in Figure S2 (supplementary data), which implies that our **A/2B16** does not follow the Schottky diode behavior. Figure 11D shows the dependence of the I-V characteristic on the scan rate. Room temperature DC-resistance and electrical conductance values of **A, B16**, and **A/2B16** at different scan rates were measured and presented in Fig. 11E,F. **B16**, **A,** and **A/2B16** have electrical resistances in the same order of magnitude, Fig. 11E, even though the complex** A/2B16** film presents higher resistance than either** A** or** B16** films. The resistance of **A/2B16** film is increased from 0.45 to 2.2 TΩ by increasing the scan rate from 0.1 to 1 V/s. From Fig. 11F, the electrical conductance of **B16** is decreased from 8.13 to 2.23 pS @ scan rate 0.1 V/s after the incorporation with Adipic acid to form the complex **A/2B16** film. By increasing the scan rate to 1 V/s, the electric conductance decreases to 0.45 pS. This behavior confirms the formation of H-bonded interactions of the prepared complex (**A/2B16**) between the adipic acid (**A**) and the lateral-chloro azopyridines (**Bn**) since the electrical conductance mainly depends on the mobility and number of charge carriers^63,64^.

### Optical spectra and energy gap calculation

**Figure 12:**
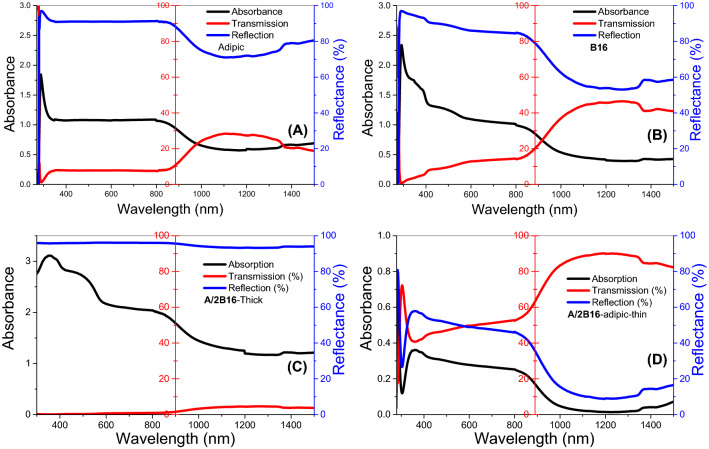
Optical spectra of (**A**) Adipic film, **A**; (**B**) Azopyridine, **B16** and (**C**) their complex **A/2B16** (**C**) thick film and (**D**) thin film.

The optical spectra of the prepared samples were investigated by Perkin Elmer spectrophotometer (Lambda 900 UV–VIS-NIR) of wavelength range from 250 to 1500 nm. Figure 12 displays the dependence of the transmittance, absorbance, and reflectance of **A**, **B16,** and **A/2B16** complex films on the wavelength. As shown in the optical spectra of adipic acid film, Fig. 12A, the transmittance of the film is almost 8% within the visible light range, which then increases exponentially in the near-IR range to reach ~ 28%@ 1100 nm. The Absorbance spectrum showed an absorption peak at 288 nm. The absorption in the UV and visible regions is higher than the absorption in the IR region. The observed high reflectance is ascribed to the use of thick film. For **B16** film, Fig. 12B, a strong absorption peak is observed at 292 nm followed by two weak absorption peaks at 345 and 385 nm, in addition to a broad peak centered around 450 nm. The transmission of the sample is slowly increased in UV and visible regions followed by the exponential increase in the near IR region to reach 45% at 1120 nm. The reflection of this film is lower than that of the acid sample.

The absorption spectra of the thick **A/2B16** complex film, Fig. 12C showed great improvement relative to either **A** or **B16** films. There are strong and broad absorption bands centered at 354, 452 and 814 nm. After that, the values of absorbance decrease exponentially to reach an almost constant value for λ > 1200 nm. The transmission of this sample is very close to zero in the UV and visible regions. This behavior confirms the formation of H-bonded interactions of the prepared complex (**A/2B16**) between the adipic acid (**A**) and the lateral-chloro azopyridines (**Bn**) since the optical properties depend mainly on the morphology and chemical composition. This could also be the reason for the red-shift of the absorption peaks of **A/2B16** relative to **B16.** This red-shift is mainly due to the size effect, where small size moderates the exciton positions and reduces spin–orbit coupling^65^. This red-shift and high absorption in UV and visible regions is a desirable feature in energy-efficient solar cells^66^. To obtain more details about the optical spectrum of **A/2B16**, complex **A/2B16** film of much lower thickness was prepared and their optical spectra are shown in Fig. 12D. This figure shows an absorption peak at 284 nm and the wide band started from 346 nm and extended to cover the visible light region. The average transmission reached 50% in the visible light region and 85% for λ > 1000 nm.

**Figure 13:**
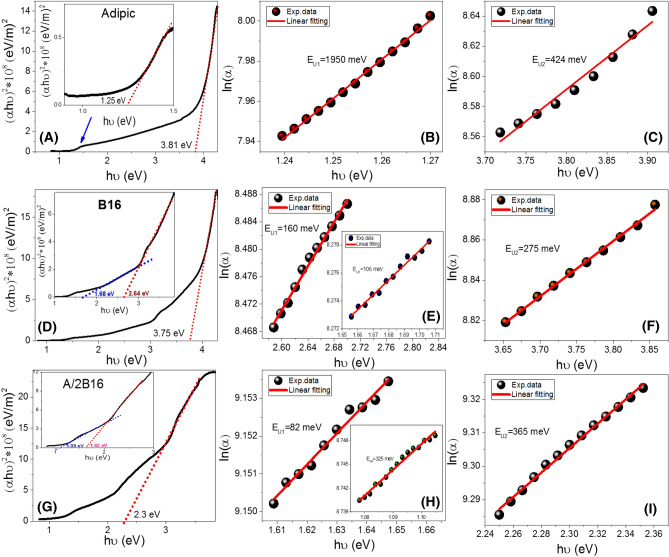
Calculation of energy gap (**A**, **D**, **G**) and Urbach energy (**B**, **C**, **E**, **F**, **H**, **I**) for individual components **A**, **B16,** and their supramolecular complex **A/2B16** films.

According to the optical absorption theorem, the correlationship between absorption coefficient, *α,* and the photon energy, *hν, is given by*^67^:

1 $$(\alpha h\nu {)}^{2/n}=A(h\nu -Eg)$$

where *h* is the Planck’s constant (6.625 × 10^−34^ J/s), *n* = 1, 4 for the direct and indirect allowed transitions, respectively, *A* is a constant, and *E*_*g*_ is the optical band-gap. The values of direct *E*_*g*_ for **A**, **B16** and **A/2B16** are obtained by extrapolating the linear portions of the plot of (*αhν*)^2^ vs. *hν* to *α* = 0 as shown in Fig. 13A,D,G. The linear parts observed in this figure indicate that the transitions are performed directly. Interestingly there are two direct band gaps for the flexible adipic acid film at 1.25 and 3.81 eV; three direct bandgaps for **B16** at 1.68, 2.64, and 3.75 eV; and three direct bandgaps for **A/2B16** at 1.09, 1.62, and 2.30 eV. The observed reduction in the bandgaps of the **A/2B16** is ascribed to the influence of the density of localized states due to formation of H-bonding interactions of the prepared complex (**A/2B16**) between the adipic acid (**A**) and the lateral-chloro azopyridines (**Bn**). This behavior is consistent to the previously reported studies^68^. The reduction of the bandgap is very important for solar energy investigations, especially photoelectrochemical hydrogen generation and solar cells.

Urbach energy (*E*_*U*_) referred to the width of the exponential absorption edge (the Urbach tail). The tails of the valence and conduction bands are ascribed to the disorder in the material^69^. The exponential dependency of the *E*_*U*_ can be determined according to the following equation^69^:

2 $${\alpha ={\alpha }_{o}exp(\frac{h\upsilon }{{E}_{U}}) \Rightarrow E}_{U}={\left[\frac{\delta (\mathit{ln}\left(\alpha \right))}{\delta \left(h\nu \right)}\right]}^{-1}$$

where *α*_*0*_ is the band tailing parameter that can be obtained by^70^;

3 $${\alpha }_{o}=\sqrt{\frac{{\sigma }_{0}(\frac{4\pi }{c})}{x\Delta E}} (6)$$

where *c* is the speed of light, *σ*_*o*_ is electrical conductivity at absolute zero, *ΔE* represents the width of the tail of the localized state in the forbidden gap. Figure 13B,C,E,F,H,I shows the plot of ln(*α*) vs. *hν* for **A**, **B16** and **A/2B16**. The values of *E*_*U*_ were obtained from the slopes of the linear fitting of these curves to be 1950 and 424 meV for adipic acid; 106, 160, and 275 meV for **B16**, and 325, 82, and 365 meV for** A/2B16,** respectively.

The molecular aggregation resulted from the terminal alkoxy chain oxygen and the –COO– moiety enhances the end-to end intermolecular interactions and side-side cohesive forces between molecules will weaken by lengthening the alkoxy group. These interactions participate with different ratios and affect the formation of mesophase^71,72^. A competitive effect resulted from both types of interactions was highly affected by the change of the confirmation of the molecule. The end-end aggregations for the formed complexes (**A/2Bn**) are more pronounced than the side-side interactions, as agreement of theoretical calculations, thus the less order nematic phase covered all lengths of designed complexes.

## Conclusion

Conical symmetrical shaped homologues of new laterally substituted 1:2 SMHBCs, bearing increased lengths of alkoxy terminal chains, were synthesized and investigated by experimental and computational approaches for solar energy investigations. The induced Fermi-bands in FT-IR spectroscopic analysis was confirm the formation of H-bonded interactions. Mesomorphic, optical and electrical properties of the present symmetrical complexes were examined by DSC, POM, Keithley measurement-source unit, and UV/Vis/IR Perkin Elmer spectrophotometer. Results revealed that all of the prepared SMHBCs exhibit an induced enantiotropic nematic mesophase. DFT simulations were established to confirm and correlate the experimental findings. Geometrical calculations showed that the increment of the terminal alkoxy chain increases the thermodynamic energy and consequently, decreases the nematic stability.

The electrical properties investigations revealed that, the **A/2B16** complex film showed nonohmic behavior with a resistance of 2.2 TΩ and electrical conductance of 2.23 pS @ scan rate 0.1 V/s, which decreased to 0.45 pS by increasing the scan rate to 1 V/s. Also, the complex **A/2B16** showed improved absorption relative to **B16**, whereas three strong and broad absorption bands centered at 354, 452, and 814 nm are detected. Also, this SMHBC showed three direct band gaps at 1.09, 1.62, and 2.30 eV with band tails of 325, 82, and 365 meV. Moreover, the optical absorption enhancement and the reduced energy-gap make the investigated long chain complex (**A/2B16**) suitable for absorption and conversion.

## Supplementary Information

Supplementary Information.
