# Supplementary material for: New nematogenic conical-shaped supramolecular H-bonded complexes for solar energy investigations
Source: Sci Rep. 2021 Sep 2;11:17622. doi: 10.1038/s41598-021-97126-5 (PMC8413331; doi:10.1038/s41598-021-97126-5)
Supplement: Supplementary file 1 — Supplementary Information. [file 41598_2021_97126_MOESM1_ESM.docx]

**Supplementary data**

**New Nematogenic Conical-Shaped Supramolecular H-bonded Complexes for Solar Energy Investigations**

**Sobhi M. Gomha^1^*, Hoda A. Ahmed ^2,3*^, Mohamed Shaban ^4^, Tariq Z. Abolibda^1^, Khalid Abdulaziz Alharbi^1^ and Hafsa H. Alalawy^2^**

1. *Chemistry Department, Faculty of Science, Islamic University in Almadinah Almonawara, Almadinah Almonawara, 42351, Saudi Arabia,* [*smgomha@iu.edu.sa*](mailto:smgomha@iu.edu.sa)
2. *Department of Chemistry, Faculty of Science, Cairo University, Cairo 12613, Egypt.*
3. *Chemistry Department, College of Sciences, Yanbu, Taibah University, Yanbu 30799, Saudi Arabia.*
4. *Department of Physics, Faculty of Science, Islamic University in Almadinah Almonawara, Almadinah, 42351, Saudi Arabia,* [*mssfadel@aucegypt.edu*](mailto:mssfadel@aucegypt.edu)

** Correspondence: Sobhi M. Gomha,* [smgomha@iu.edu.sa](mailto:smgomha@iu.edu.sa)*; Hoda A. Ahmed,* [*ahoda@sci.cu.edu.eg*](mailto:ahoda@sci.cu.edu.eg)

1. **Materials**

Adipic acid was obtained from Merck (Germany). N,N'- dicyclohexylcarbodiimide (DCC) and 4-dimethylaminopyridine (DMAP) were purchased from Aldrich (Wisconsin, USA). All solvents used are pure grade and purchased from Aldrich (Wisconsin, USA).

**2. Characterizations and instrumentation**

Purity of all prepared compounds were checked with thin-layer chromatography using TLC-sheets coated with silica gel (E. Merck), whereby single spots were detected by a UV-lamp.

Molecular formulae of the prepared base compounds (**Bn**) were confirmed via elemental analyses, infrared, Mass spectra and ^1^H-NMR spectroscopy. The results agreed with the proposed structures and with those reported in the literature [1].

4-(2-(Pyridin-4-yl)diazenyl-3-chlorophenyl) 4-decyloxybenzoate, **B10**

Yield: 97.2%; mp 98.8°C, FTIR (ύ, cm−1): 2918–2855 (CH2 stretching), 1725 (C=O), 1590(C=C), 1468 (C–O Asym), 1412 (N = N), 1249 (C–O Sym). 1H NMR (400MHz, CDCl3): δ/ppm: 0.69 (t, 3H, CH3(CH2)7CH2CH2, J = 6.6 Hz), 1.21–1.41 (m, 14H, CH3(CH2)7 CH2CH2), 1.73–1.81 (m, 2H, CH3(CH2)7CH2CH2), 3.98 (m, 2H, CH3(CH2)7CH2CH2), 8.29 (d, 0.89 H, J = 8.3 Hz, Ar–H, Z-isomer), 8.16 (d, 2H, J = 8.2 Hz, Ar–H, E-isomer), 8.08 (d, 0.44H, J = 8.1 Hz, Ar–HZ-isomer), 7.88 (d, 1H, J = 8.9 Hz, Ar–H, E-isomer), 7.55 (d, 1H, J = 8.3 Hz, Ar–H, E-isomer), 7.35–7.27 (m, 4.4H, Ar–H), 7.01 (m, 2.7H, Ar–H). 13C NMR (101 MHz, CDCl3) δ:ppm: 164.17, 163.97, 153.94, 151.62, 148.11, 147.63, 137.02, 132.44, 127.48, 124.18, 121.19, 120.59, 119.96, 118.58, 68.43, 31.91, 29.71, 29.22, 29.08, 29.05, 25.99, 22.70, 15.29, 14.14. Elemental analyses: Found (Calc.): C, 68.06 (68.07); H, 6.51 (6.53); N, 8.48 (8.51); Cl, 7.15 (7.18).

TA Instruments Co. Q20 Differential Scanning Calorimeter (DSC; USA) were using for calorimetric measurements. The DSC was calibrated using the melting temperature and enthalpy of indium and lead. DSC investigation was carried out for small samples (2–3 mg) placed in aluminum pans. All measurements were achieved at a heating rate of 10°C/min in inert atmosphere of nitrogen gas (30 ml/min) and all transition recorded from the second heating scan.

Transition temperatures for 1:2 complexes (**A/2Bn**), were determined by DSC, and the types of the mesophase identified by a standard polarized light microscope (PLM, Wild, Germany) attached with Mettler FP82HT hot stage. The temperature is measured by thermocouple attached to the temperature controller. Measurements were made twice and the results have accuracy in transition temperature within ± 0.2°C.

**3*.* Computational charactrizations**

Optimized geometries of complexes were calculated using the Gaussian 09 package [2]. Calculations were carried out using DFT approach at B3LYP/6-31G(d,p) level of theory. Frequency calculations showed that all structures were stationary points in the geometry optimization with no imaginary frequency. The B3LYP method was performed for optimization of complexes **(A/2Bn)** where the geometries were optimized by minimizing the energies with respect to all geometrical parameters without imposing any molecular symmetry constraints. Ionization energy (**I.E**) is calculated as the negative of the energy of the HOMO. . ΔE, the energy gap, is calculated as the difference between ELUMO and EHOMO.

**Table S1** H-bond length and dihedral angle calculated for the optimized structure of the four complexes at B3LYP/6-31g(d,p) level of theory.

| Complex | H-bond 1, Å | H-bond 2, Å | Dihedral angle, ^⁰^  C-C-C-C |
| --- | --- | --- | --- |
| **A/2B8** | 1.74788 | 1.82597 | -68.01284 |
| **A/2B10** | 1.74633 | 1.82538 | -68.06245 |
| **A/2B12** | 1.75101 | 1.82395 | -70.40207 |
| **A/2B16** | 1.74996 | 1.82479 | -69.74443 |


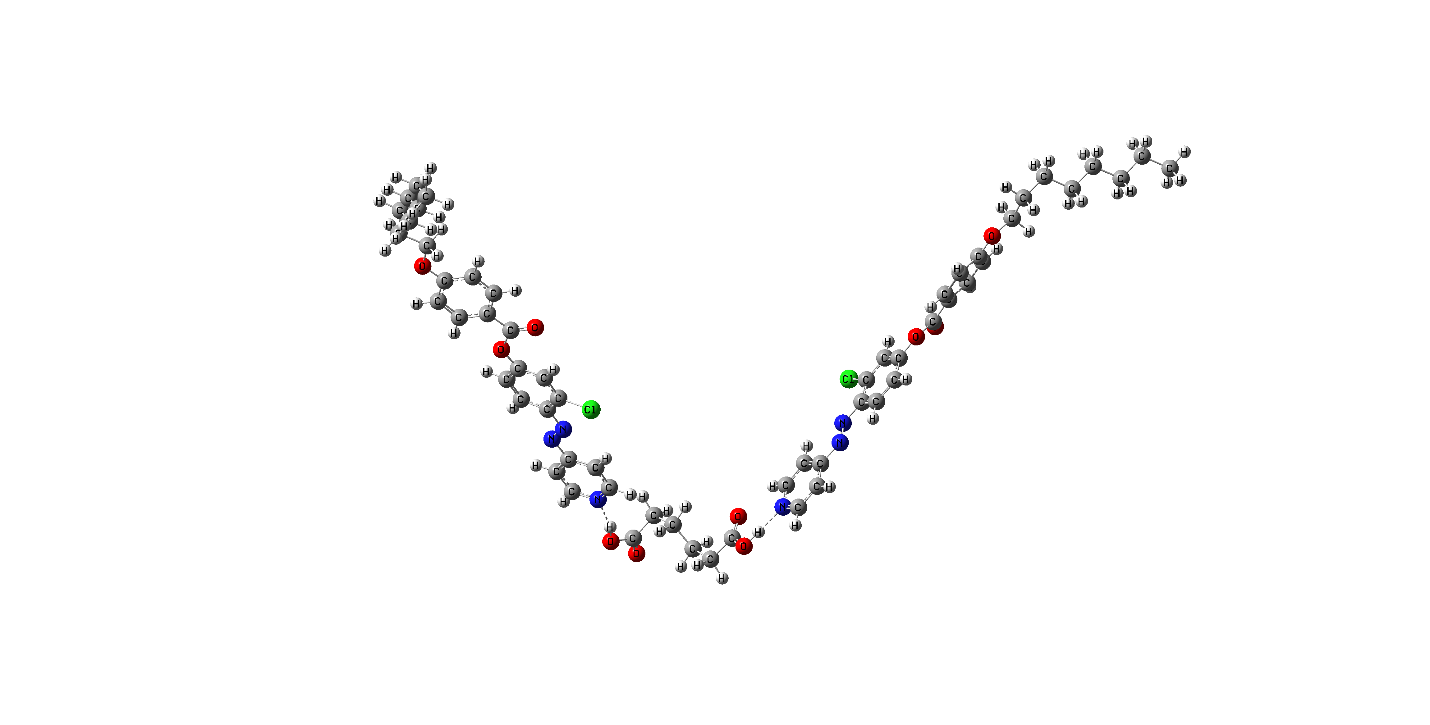


68.01284⁰

1.82597

1.74788

**Figure S1:** H-bond length, in Å and dihedral angle for the optimized structure of the **A/2B8** complex calculated at B3LYP/6-31g(d,p) level of theory.

**Figure S2:** Log(I) –V^1/2^ characteristics of adipic acid (**A)**, **B16** and **A/2B16** films.

References:

[1] Hagar M, Ahmed H, Alhaddad O. Experimental and theoretical approaches of molecular geometry and mesophase behaviour relationship of laterally substituted azopyridines. Liq Cryst. 2019;46:1440–1451.

[2] Frisch, M. J. et.al. 1998, Gaussian 98, revision A.7, Gaussian Inc., Pittsburgh, PA.
